# Scarlet Fever

**Published:** 1894-07-14

**Authors:** 


### MEDICAL PROGRESS.

### SCARLET FEVER.

The occurrence of a second attack of scarlet fever in the same patient having been mentioned last autumn in the *British Medical Journal*, a considerable number of correspondents have recorded similar instances. These cannot have all been due to mistakes in diagnosis, and the probability is that second attacks or relapses are more common than is usually supposed. Thus H. B. Beatty, R.N., records two cases,[1] one six weeks and the other eleven months after the original attack. T. D. Wynne[2] notes no less than ten cases out of 600 consecutive patients in the Nottingham isolation hospital. These may be indeed classed as relapses, as they occurred in the 2nd, 3rd, 4th, 5th, 6th, and 7th weeks after the original attacks. Both primary and secondary outbreaks were, as a rule, mild—both were marked by a rash, rise of temperature, and sore throat. Another with three weeks' interval is reported by A. H. Walker,[3] one with five weeks' interval from Penygroes, North Wales,[4] and one with three weeks from Tenbury.[5] In the two last it was noted that peeling of the skin and the second rash were to be seen together. Mr. Greenwood had an attack repeated after three months, desquamation following each outbreak.[6] Three weeks' interval was seen in a case from North Carolina, which is reported minutely in the *American Medical and Surgical Bulletin*.[7] The same interval occurred in an eighteenth case, which was associated with one in which measles occurred when the fever attack was passing off.[8]

While some of these were new attacks, others seem to be rather instances of secondary pyrexia, such as a case described by Clarke,[9] where the temperature fell to 100 deg. on the fourth day, and rose again on the sixth to 102·8 deg., and did not become normal till the sixteenth. In some parts of the body there was double desquamation. The author also quotes several instances of prolonged or revived pyrexia in scarlet fever, lasting for two or more weeks, from Eustace Smith, Long Fox, Thomas, and Henoch. Of course such cases are distinct from those where otitis or other complications were present. The subject is discussed by Bouveret, and an account given of three cases where high temperatures followed an apyretic period. Bouveret and Clarke incline to the view that the phenomenon is due to a toxine of scarlatinal origin,[10] but possibly, indeed, to a secondary pyæmic infection to which the chart of Clarke's case points strongly. It should be remarked that almost all the instances referred to above showed fresh throat inflammation, and though the temperatures are not fully given, there is reason to think that an apyretic period preceded the second attack, except in Clarke's case.

A different type of complication is recorded by D. Macartney.[11] Pus formed in the mastoid process, and in the joints of the right hip, elbow, and great toe; necrosis at the end of the left clavicle, and in the mastoid processes followed, but the patient, a girl of six, recovered thoroughly. Similar cases are mentioned by Henoch and West.

Outbreaks due to infected milk are discussed by Chalmers, from Glasgow,[12] and S. Wilson, from Hastings.[13] In the latter the cattle were found to be suffering from some febrile disturbance, and no other source was detected.

Dr. Clement Dukes started an interesting discussion on the diagnosis of epidemic roseola and scarlatina.[14] He has never seen the former develop into the latter, but the trouble and expense caused by the difficulty of distinguishing the two diseases from each other and from measles is a serious matter. Three other eruptions have also a close resemblance to epidemic roseola, viz., copaiba rash, roseola simplex, and that produced by handling certain British caterpillars, but in none of these are the lymphatic glands enlarged.

The points of difference between scarlatina and epidemic roseola are: Roseola has usually an incubation period of nine to twenty-one days; it has usually no initial malaise, and occurs generally in spring and summer, while scarlatina is chiefly found in autumn and winter. The rash is more rosy, and spreads over the body more quickly than in scarlatina, and the skin is less hot to the touch. The axillary, posterior cervical and inguinal glands are enlarged and tender, the conjunctivœ suffused, the desquamation very variable, but always in fine scales, the tongue is never thickly coated, and infectivity is very high in the earliest stage (unlike that of scarlatina), but disappears in two or three weeks. The desquamation, too, comes to an end in a week or two. Dr. Bridgman,[15] on the other hand, narrates an epidemic which Dr. Dukes himself diagnosed as E. Roseola, where the peeling was in large scales and lasted six weeks, and otitis and pericarditis were found as sequelæ. He insists that the posterior cervical glands are enlarged also in the early stage of scarlatina, but confesses rotheln patients alone complain of their pain and tenderness. Donkin[16] disbelieves in the possibility of placing at present as a distinct disease "these cases which are at first like scarlatina, but which turn out not to be so." Diagnosis is most difficult, and the enlargement of the cervical glands is uncertain in what is usually called E. Roseola, and is frequent in scarlatina. Practically, if we have a number of "patients suffering from a roseolous type of disease, most of whom have previously had scarlatina or measles, we can then, and then alone, exclude these diseases."

[1] B.M.J., March 17, 1894; [2] B.M.J., March 17, 1894; [3] B.M.J., March 24, 1894; [4] B.M.J., Feb. 24, 1894; [5] B.M.J., Feb. 17th, 1894; [6] B.M.J., Feb. 24. 1894; [7] Am. Med. and S. Bulletin, Mar. 15, 1894; [8] B.M.J., Mar. 10, 1894; [9] Quarterly Med. Jour., April, 1894; [10] Revue de Med., April, 1892; [11] Glasgow Med. Jour., April, 1894; [12] B.M.J., Feb. 24, 1894; [13] B.M.J., April 14, 1894; [14] Lancet, March 31, 1894; [15] Lancet, April 21, 1894; [16] Lancet, May 19, 1894.

